# The tumor immune microenvironment transcriptomic subtypes of colorectal cancer for prognosis and development of precise immunotherapy

**DOI:** 10.1093/gastro/goaa045

**Published:** 2020-09-14

**Authors:** Yun-Qiang Tang, Tu-Feng Chen, Yan Zhang, Xiao-Chen Zhao, Yu-Zi Zhang, Guo-Qiang Wang, Meng-Li Huang, Shang-Li Cai, Jing Zhao, Bo Wei, Jun Huang

**Affiliations:** Department of Hepatic-biliary Surgery, Affiliated Cancer Hospital and Institute of Guangzhou Medical University, Guangzhou, Guangdong, P. R. China; Department of Gastrointestinal Surgery, The Third Affiliated Hospital of Sun Yat-sen University, Guangzhou, Guangdong, P. R. China; Jiangsu Cancer Hospital & Jiangsu Institute of Cancer, Research & The Affiliated Cancer Hospital of Nanjing Medical University, Nanjing, Jiangsu, P. R. China; The Medical Department, 3D Medicines Inc., Shanghai, P. R. China; The Medical Department, 3D Medicines Inc., Shanghai, P. R. China; The Medical Department, 3D Medicines Inc., Shanghai, P. R. China; The Medical Department, 3D Medicines Inc., Shanghai, P. R. China; The Medical Department, 3D Medicines Inc., Shanghai, P. R. China; The Medical Department, 3D Medicines Inc., Shanghai, P. R. China; Department of Gastrointestinal Surgery, The Third Affiliated Hospital of Sun Yat-sen University, Guangzhou, Guangdong, P. R. China; Department of Colorectal Surgery, The Sixth Affiliated Hospital of Sun Yat-sen University, Guangzhou, Guangdong, P. R. China; Guangdong Provincial Key Laboratory of Colorectal and Pelvic Floor Diseases, the Sixth Affiliated Hospital, Sun Yat-sen University, Guangzhou, P. R. China; Guangdong Institute of Gastroenterology, Guangzhou, P. R. China

**Keywords:** colorectal cancer, immune dysfunction, immune exclusion, prognosis

## Abstract

**Background:**

Biomarkers based on immune context may guide prognosis prediction. T-cell inactivation, exclusion, or dysfunction could cause unfavorable tumor microenvironments, which affect immunotherapy and prognosis. However, none of the immuno-biomarkers reported to date can differentiate colorectal-cancer (CRC) patients. Thus, we aimed to classify CRC patients according to the levels of T-cell activation, exclusion, and dysfunction in the tumor microenvironment.

**Methods:**

RNAseq data of 618 CRC patients from The Cancer Genome Atlas and microarray data of 316 CRC patients from Gene Expression Omnibus were analysed using the Tumor Immune Dysfunction and Exclusion algorithm. Unsupervised clustering was used to classify patients.

**Results:**

Based on the expression signatures of myeloid-derived suppressor cells, cancer-associated fibroblasts, M2-like tumor-associated macrophages, cytotoxic T-lymphocytes, and PD-L1, all patients were clustered into four subtypes: cluster 1 had a high level of immune dysfunction, cluster 2 had a low level of immune activation, cluster 3 had intense immune exclusion, and cluster 4 had a high level of immune activation and a moderate level of both dysfunction and exclusion signatures. Compared with cluster 1, the hazard ratios and 95% confidential intervals for overall survival were 0.63 (0.35–1.13) for cluster 2, 0.55 (0.29–1.03) for cluster 3, and 0.30 (0.14–0.64) for cluster 4 in multivariate Cox regression. Similar immune clustering and prognosis patterns were obtained upon validation in the GSE39582 cohort. In subgroup analysis, immune clustering was significantly associated with overall survival among stage I/II patients, microsatellite stable/instability-low patients, and patients not treated with adjuvant therapy.

**Conclusions:**

Our findings demonstrated that classifying CRC patients into different immune subtypes serves as a reliable prognosis predictor and may help to refine patient selection for personalized cancer immunotherapy.

## Introduction

Multiple steps are required to mount an immune response against tumor cells, including antigen presentation, T-cell activation and expansion, and prolonged cytotoxic T-lymphocytes (CTLs) activity [1]. During this process, two types of tumor immune microenvironments (TIMEs) could prevent cytotoxic T-cells from killing tumor cells: T-cell exclusion and T-cell dysfunction [2]. The former is characterized by a low level of cytotoxic T-cell infiltration in the tumor center where tumor cells are surrounded by a large number of immunosuppressive cells that physically exclude cytotoxic T-cells at invasive margins [3]. There are mainly three kinds of immunosuppressive cells involved: tumor-associated M2-type macrophages (TAM.M2), myeloid-derived suppressor cells (MDSCs), and cancer-associated fibroblasts (CAFs) [3]. In the case of T-cell dysfunction, tumor cells evade immune surveillance even with a massive influx of cytotoxic T-cells into the tumor center because these T-cells are usually dysfunctional. For example, immune-checkpoint molecules expressed on T-cells such as programmed cell death protein 1 (PD-1), cytotoxic T-lymphocyte protein 4 (CTLA-4), and lymphocyte-activation gene 3 (LAG3) may inhibit T-cell function [4]. Accumulating evidence has suggested that subtyping patients based on TIME could inform prognosis prediction. In colorectal cancer (CRC), studies have shown that the type, density, and location of immune cells within tumors could predict survival more accurately than the TNM stage system [5] and microsatellites instable (MSI) status [6]. T-cell activity, as measured by the density of CD3^+^ and CD8^+^ T-lymphocytes at the margins and center of tumors, acts as a prognostic biomarker for disease-free survival and overall survival (OS) in early-stage CRC patients independently of TNM stage and MSI status [7, 8]. Besides, the expression of genes related to the immunosuppressive cells or T-cell exclusion signatures were reported to be associated with poor prognosis in CRC [9, 10]. Furthermore, a recent meta-analysis indicated that a high level of PD-1 ligand 1 (PD-L1) expression might be an indicator of a poor prognosis in CRC patients [11]. Collectively, CRC patients may be classified into subsets with distinct TIMEs taking into account T-cell activation, exclusion, and dysfunction to predict prognosis.

Several TIME biomarkers have been developed based on the expression of immune-cell signatures or genes involved in immune activation to predict prognosis in CRC patients, including the immune-risk score from 22 types of immune-cell signatures [12], consensus molecular subtypes from the integration of six subtyping platforms [13], and coordinated immune-response clusters from 24 immune genes [14]. However, these biomarkers may only classify patients into groups with strong or weak immune activities, but could not further dissect the patients with weak immune response into subsets characterized by T-cell dysfunction, inadequate T-cell activation, or T-cell exclusion. Jiang *et al*. [2] developed an algorithm called ‘Tumor Immune Dysfunction and Exclusion’ (TIDE) to estimate the level of T-cell dysfunction as well as T-cell exclusion featuring TAM.M2, MDSC, and CAF. The TIDE signature was shown to be inversely associated with response to anti-PD-1/anti-CTLA4 in melanoma patients [2].

With the goal of classifying CRC patients based on the dynamics between T-cell activation, exclusion, and dysfunction, we profiled the expression of *CD8A*, *CD8B*, *GZMA*, *GZMB*, and *PRF1* for T-cell activation, TIDE developed by Jiang *et al*. [2] to estimate TAM.M2, MDSC, and CAF signatures, as well as PD-L1 gene expression to represent T-cell dysfunction in two independent cohorts. Here, we integrated the above-mentioned five signatures to define immune subtypes that may act as a prognostic biomarker.

## Materials and methods

### Data source

We obtained the whole-exome sequencing and RNAseq data (IlluminaHiSeq) of 618 CRC patients from The Cancer Genome Atlas (TCGA) (https://portal.gdc.cancer.gov/) as the discovery cohort and mRNA microarray data (Affymetrix Human Genome U133 Plus 2.0 Array) of 316 CRC patients without adjuvant treatment from GSE39582 (https://www.ncbi.nlm.nih.gov/geo/query/acc.cgi? acc=GSE39582) as the validation cohort. DNA and RNA extraction, library preparation, sequencing, quality control, and subsequent data processing were performed as described previously [15]. MSI status was evaluated by the TCGA consortium using a panel of four mononucleotide repeats (BAT25, BAT26, BAT40, and TGFBRII) and three dinucleotide repeats (D2S123, D5S346, and D17S250) [16]. Tumors were classified as MSI-H (≥40% of markers altered), MSI-L (<40% of markers altered), and MSS (no marker altered). The mutation definition for a specific gene in the TCGA cohort was based on the alterations listed in the OncoKB database (http://oncokb.org/). The consensus molecular subtypes (CMS) data for the TCGA cohort were downloaded from the supplemental materials in corresponding literature [17]. The samples in the GSE39582 cohort were hybridized with Affymetrix HG-U133 Plus 2.0 (GEO accession number GPL570) microarrays. The raw ‘CEL’ files of the microarray data were downloaded and normalized using the MAS5 method [18]. Detailed information regarding clinical characteristics, genetic mutations, and dMMR status were described in the original paper [19].

### mRNA-expression profiling analysis and cell-signatures estimation

The genes related to immune context included T-effector, interferon gamma (IFNG)-associated genes, and immune-checkpoint genes. The immune gene list was reported previously [20]. The mRNA-expression levels for the TCGA cohort were quantified using the Fragments Per Kilobase of transcript per Million fragments mapped (FPKM) approach. The data were log2-transformed before analysis. Based on the immune-response process, we extracted three categories of signatures for the clustering analysis: T-cell activation, T-cell exclusion, and T-cell dysfunction. T-cell-activation levels were estimated using CTL-infiltration signatures, which were calculated from the average expression of *CD8A*, *CD8B*, *GZMA*, *GZMB*, and *PRF1*. T-cell exclusion was measured by signatures of MDSC, CAF, and TAM.M2. The correlation matrix of immune-checkpoint-related-genes expression showed that *PD-L1* expression was significantly correlated with other immune-checkpoint genes (Supplementary Figure 1). Therefore, *PD-L1* expression was selected to represent the T-cell dysfunction level. Finally, CTL, MDSC, CAF, TAM.M2, and PD-L1 were included in the unsupervised clustering. The signatures of MDSC, CAF, TAM.M2, T-cell dysfunction, and exclusion were estimated using mRNA-expression data on the website http://tide.dfci.harvard.edu/.

### Statistical analyses

Student’s *t*-test for normally distributed data and Mann–Whitney *U* test for non-normally distributed data were used to determine the differences between two groups. Differences among three or more groups were determined by the Kruskal–Wallis test. Chi-square test or Fisher exact test was used to test the difference between groups for categorical variables. *P*-values were adjusted using the Benjamini–Hochberg method. The clusters of immune signatures were obtained from unsupervised hierarchical cluster analysis. Hazard ratio was determined through multivariate Cox regression. Proportional-hazards assumption was tested before the Cox regression. Pearson-correlation coefficient was used to estimate correlation among various genes expression. The performance analytics R package was used to draw the correlation plot. All reported *P*-values were two-tailed and a *P*-value of <0.05 was considered significant. All the analyses were performed using R 3.5.2 software (Murray Hill, NJ, USA).

## Results

### Unsupervised clustering analysis for immune signatures in the TCGA cohort

**Figure 1.**
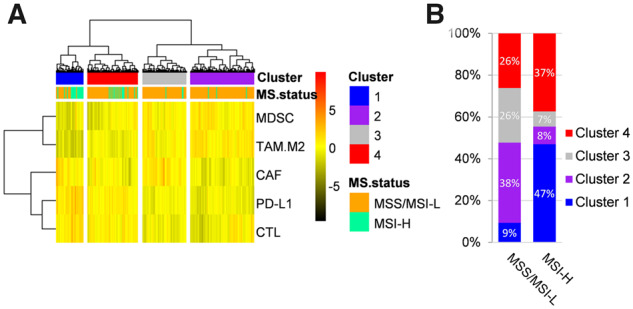
Unsupervised clustering analysis for immune signatures in TCGA cohort. (A) Heat map of unsupervised clustering analysis. (B) The proportion of clusters in MSS/MSI-L and MSI-H patients. TCGS, The Cancer Genome Atlas; MS.status, status of microsatellite instability; MSI-H, microsatellite instability high; MSI-L, microsatellite instability-low; MSS, microsatellite stable; CTLs, cytotoxic T-lymphocytes; CAF, cancer-associated fibroblasts; MDSCs, myeloid-derived suppressor cells; TAM.M2, the M2 subtype of tumor-associated macrophages (TAMs).

Following unsupervised clustering analysis that included CTL, MDSC, CAF, TAM.M2, and PD-L1, four clusters were identified with distinct immune signature patterns (Figure 1A). The majority of MSI-H patients were found in clusters 1 (47%) and 4 (37%) (Figure 1), whereas MSS/MSI-L patients were distributed among clusters 1, 2, 3, and 4 at 9%, 38%, 26%, and 26%, respectively (Figure 1B).

### Comparison of immune-genes expression and signatures across various clusters

**Figure 2.**
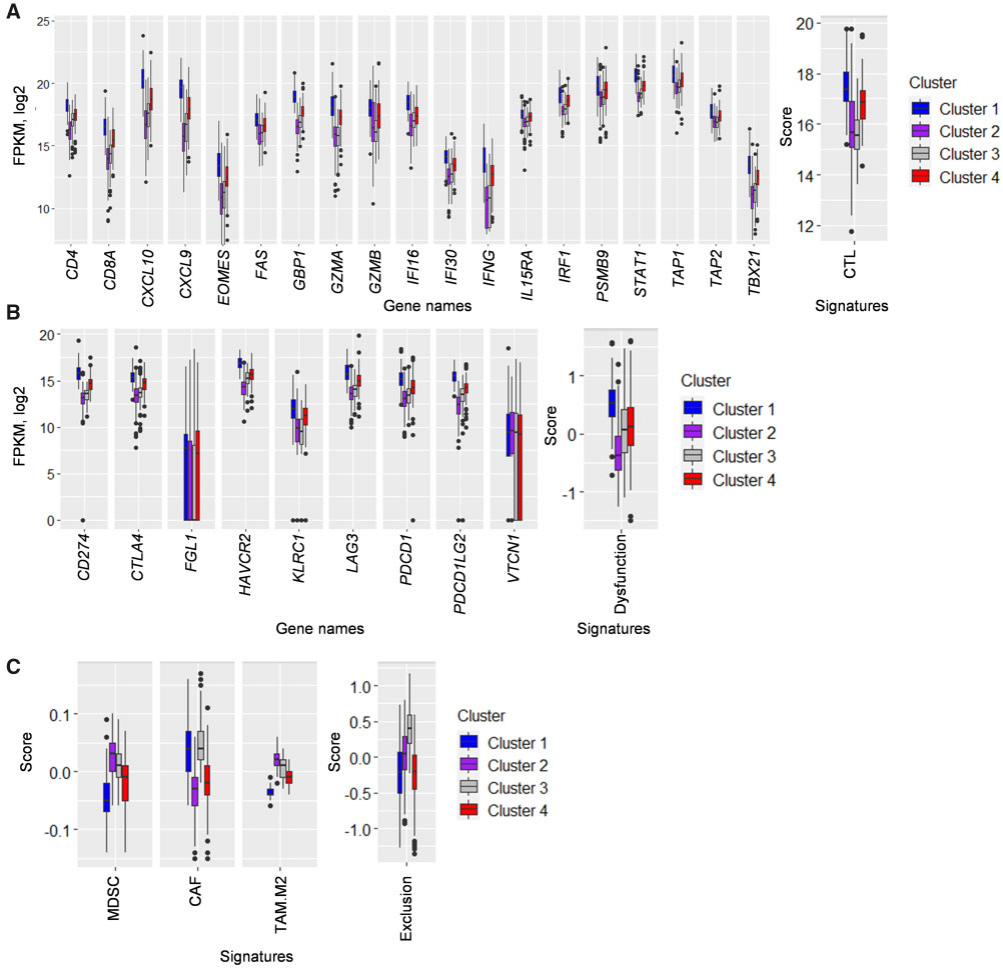
Comparison of immune genes or signatures across various clusters. (A) The association of immune-activation-related gene mRNA-expression levels and CTL signature scores with clusters, all *P*-values <0.001. (B) The association of immune-checkpoint-related gene mRNA-expression levels and T-cell dysfunction signature scores with clusters, all *P*-values <0.001 (except for VTCN1, *P* = 0.900). (C) The association of T-cell infiltration-inhibition-related immune-cell signature scores with clusters, all *P*-values <0.001. All the *P*-values were adjusted using the Benjamini–Hochberg method. CTLs, cytotoxic T-lymphocytes; CAFs, cancer-associated fibroblasts; MDSCs, myeloid-derived suppressor cells; TAM.M2, the M2 subtype of tumor-associated macrophages (TAMs).

In order to explore the TIME characteristics of each cluster, the expression levels of genes involved in immune activation along with key immune signatures were compared across clusters. Cluster 1 and clusters 2 and 3 were observed to have the highest and the lowest levels of expression of immune-activation-related genes, respectively, whereas cluster 4 fell in between them with a moderate expression level (all adjusted *P* < 0.001, Figure 2A). A similar expression pattern was also observed for immune-checkpoint-encoding genes: cluster 1 > cluster 4 > clusters 2/3 (all adjusted *P* < 0.001 except for *VTCN1*, *P* = 0.900, Figure 2B). The four clusters also showed different levels of the three T-cell-exclusion signatures. MDSC and TAM.M2 were most abundant in cluster 2 and least abundant in cluster 1, whereas CAF was found at the highest level in clusters 1 and 3 and the lowest level in cluster 2. Cluster 4 tumors appeared to have a moderate number of all three signatures (all adjusted *P* < 0.001, Figure 2C). When TIDE signatures (T-cell exclusion and dysfunction) were combined with immune-activation-related genes to further characterize the subtypes, cluster 1 showed potent T-cell infiltration but the highest level of immune-checkpoint expression, indicating that CTL function in the TIME of this group is likely to be suppressed by immune checkpoints and therefore it is termed as ‘immune-dysfunctional’. Cluster 4 exhibited the second-highest level of CTL infiltration and moderate levels of immune checkpoints and exclusion markers, and is thus postulated to be ‘immune-amenable’. Clusters 2 and 3 both had the lowest levels of CTL, but cluster 3 also featured the highest exclusion-marker expression, suggesting strong T-cell exclusion, while cluster 2 resembled ‘cold tumors’ in terms of weak immune-activation profiles and low immune-checkpoint levels, so clusters 2 and 3 may be referred to as ‘immune-inactive’ and ‘immune-excluded’, respectively. Based on their immune context, cluster 4 is expected to have the best prognosis among all subtypes.

### Survival analysis for the four clusters and OS

**Figure. 3.**
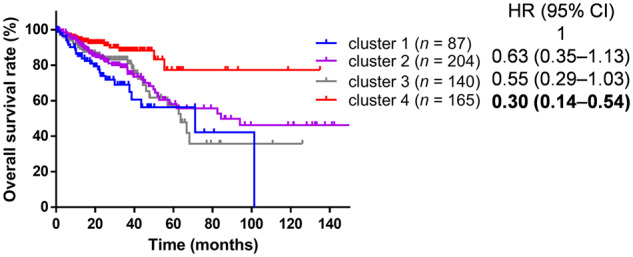
Kaplan–Meier survival analysis for four clusters and overall survival. Hazard ratios were adjusted for age, pathologic stage, tumor side, MS status, *BRAF* mutation, and *PIK3CA* mutation.

Among the baseline characteristics, age, tumor stage, tumor site, MSI-H phenotype, *BRAF* mutation, and *PIK3CA* mutation were significantly different across the four clusters (**Table 1**) and were adjusted in the following survival analysis. Compared with cluster 1, the hazard ratios (HRs) and 95% confidential intervals (CIs) for OS were 0.63 (0.35–1.13) for cluster 2, 0.55 (0.29–1.03) for cluster 3, and 0.30 (0.14–0.64) for cluster 4 (Figure 3), indicating that cluster 4 had the best prognosis among all subtypes. This is consistent with the prediction based on the immune subtyping as described above.

**Table 1.** The basic characteristics of patients across four clusters

Characteristics	Cluster 1 (*n* = 87)	Cluster 2 (*n* = 204)	Cluster 3 (*n* = 140)	Cluster 4 (*n* = 165)	*P*-value
Immune type	Dysfunction	Inactivation	Exclusion	Moderation	
Age, years	69 (62–79)	66 (54–74)	68 (57–74)	68 (59–76)	0.040
Males	45 (51.7)	107 (52.5)	76 (54.3)	94 (57.0)	0.910
Stage I + II	50 (57.5)	110 (53.9)	53 (37.9)	103 (62.4)	<0.001
Venous invasion	20 (23.0)	40 (19.6)	38 (27.1)	27 (16.4)	0.240
Tumor side					0.002
Left	32 (36.8)	123 (60.3)	89 (63.6)	83 (50.3)	
Right	42 (48.3)	66 (32.4)	40 (28.6)	63 (38.2)	
Transverse	6 (6.9)	10 (4.9)	5 (3.6)	15 (9.1)	
Lymph node examined counts <2	10 (11.5)	24 (11.8)	11 (7.9)	16 (9.7)	0.720
MSI-H	39 (44.8)	7 (3.4)	6 (4.3)	31 (18.8)	<0.001
TMB	184 (95–1,166)	105 (77–136)	93 (72–125)	105 (84–205)	<0.001
*TP53* mutation	29 (33.3)	70 (34.3)	50 (35.7)	50 (30.3)	0.84
*BRAF* mutation	12 (13.8)	2 (1.0)	3 (2.1)	9 (5.5)	<0.001
*KRAS* mutation	19 (21.8)	59 (28.9)	39 (27.9)	26 (15.8)	0.02
*NRAS* mutation	2 (2.3)	11 (5.4)	5 (3.6)	3 (1.8)	0.310
*PIK3CA* mutation	16 (18.4)	22 (10.8)	10 (7.1)	7 (4.2)	0.003
*POLE* mutation	1 (1.1)	1 (0.5)	1 (0.7)	4 (2.4)	0.380
CMS					<0.001
CMS1	35 (40.2)	6 (2.9)	5 (3.6)	27 (16.4)	
CMS2	2 (2.3)	108 (52.3)	44 (31.4)	55 (33.3)	
CMS3	2 (2.3)	42 (20.6)	9 (6.4)	19 (11.5)	
CMS4	38 (43.7)	6 (2.9)	59 (42.1)	33 (20.0)	
Unknown	6 (6.9)	13 (6.4)	17 (12.1)	21 (12.7)	

Continuous variables are presented as median (interquartile) and categorical variables are presented as counts (percentage). Twenty-two patients without survival information are excluded in this table.

MSI-H, microsatellite instability high; TMB, tumor mutation burden; CMS, consensus molecular subtypes.

**Figure 4.**
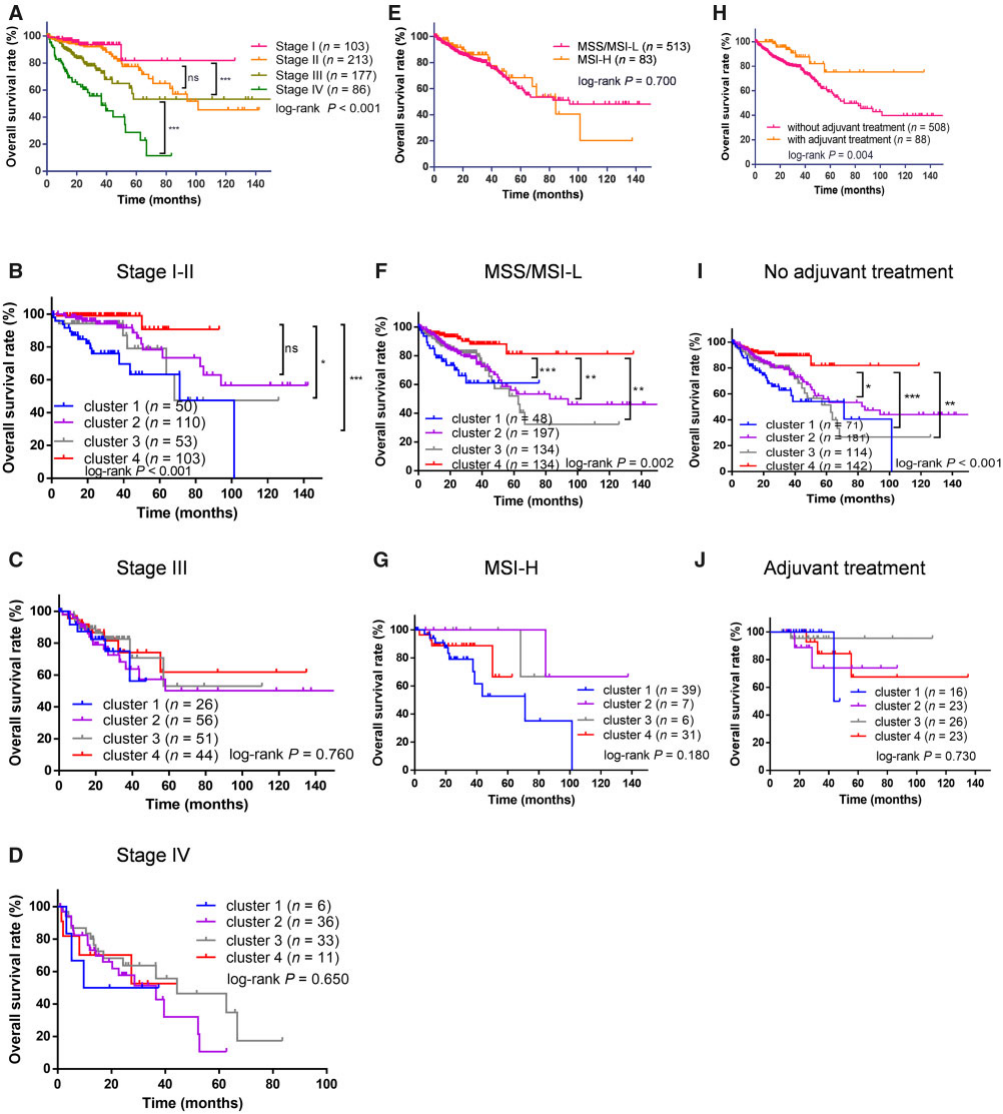
Kaplan–Meier survival analysis for subgroups analyses. (A) The comparison of OS across various stages. (B) The comparison of OS across clusters within stage I–II patients. (C) The comparison of OS across clusters within stage III patients. (D) The comparison of OS across clusters within stage IV patients. (E) The comparison of OS between MSS/MSI-L and MSI-H. (F) The comparison of OS across clusters within MSS/MSI-L patients. (G) The comparison of OS across clusters within MSI-H patients. (H) The comparison of OS between patients with and without adjuvant treatment. (I) The comparison of OS across clusters within patients without adjuvant treatment. (J) The comparison of OS across clusters within patients with adjuvant treatment. ns, not significant, *P < 0.050, **P < 0.010, ***P < 0.001.

Subgroup analyses were conducted for pathological stage, MSI status, and adjuvant treatment. The OS was inversely associated with higher pathological stage (log-rank *P* < 0.001, Figure 4A). Within stage I/II patients, cluster 4 has significantly longer OS than clusters 1–3 (log-rank *P* < 0.001, Figure 4B). For stage III/IV, no significant difference in OS was observed across the four clusters (Figure 4C and D). OS was similar between MSS/MSI-L and MIS-H patients (log-rank *P* = 0.700, Figure 4E). Cluster 4 had significantly longer OS than the other three clusters among MSS/MSI-L patients (log-rank *P* = 0.002, Figure 4F), but not in the MSI-H subset (log-rank *P* = 0.180, Figure 4G). Compared with patients having received adjuvant treatment, patients not being subjected to adjuvant therapy had significantly longer OS (log-rank *P* = 0.004, Figure 4H). For patients not treated in the adjuvant setting, cluster 4 showed significantly longer OS than the other three clusters (log-rank *P* < 0.001, Figure 4I), but this pattern was not observed among patients having received adjuvant treatment (log-rank *P* = 0.730, Figure 4J).

### Validation of association between the four clusters and OS in the GEO cohort

**Figure 5.**
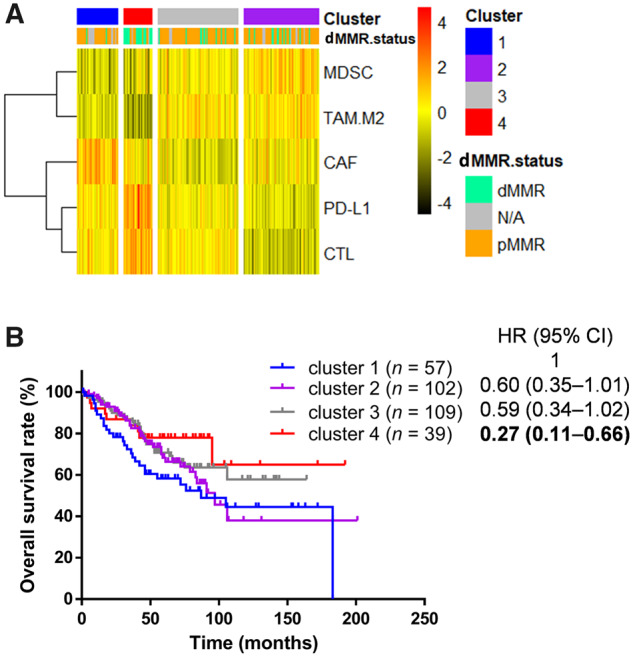
Validation of the association between immune signature and prognosis in GEO cohort. (A) Unsupervised clustering analysis for immune signatures in the GEO cohort. (B) Kaplan–Meier survival analysis for four clusters and overall survival. Hazard ratios were adjusted for age, pathologic stage, tumor location, MMR status, and *BRAF* mutation. Because 9 of 316 patients lacked OS data, only 307 patients were included in this analysis. CTLs, cytotoxic T-lymphocytes; CAFs, cancer-associated fibroblasts; MDSCs, myeloid-derived suppressor cells; TAM.M2, the M2 subtype of tumor-associated macrophages (TAMs).

To validate the prognostic effect of immune subtyping, unsupervised clustering was conducted on the same TIDE signatures and immune-activation-related genes as described above for the GSE39582 cohort, and four clusters with immune characteristics similar to those obtained from the TCGA cohort were identified: an immune-dysfunctional cluster, an immune-inactive cluster, an immune-excluded cluster, and an immune-amenable cluster (Figure 5A). In line with the prognosis pattern observed in the TCGA cohort, clusters 2, 3, and 4 displayed HRs and 95% CIs of 0.60 (0.35–1.01), 0.59 (0.34–1.02), and 0.27 (0.11–0.66), respectively, for OS compared with cluster 1 (Figure 5B).

### Proposed scheme for stratification of CRC patients

**Figure 6.**
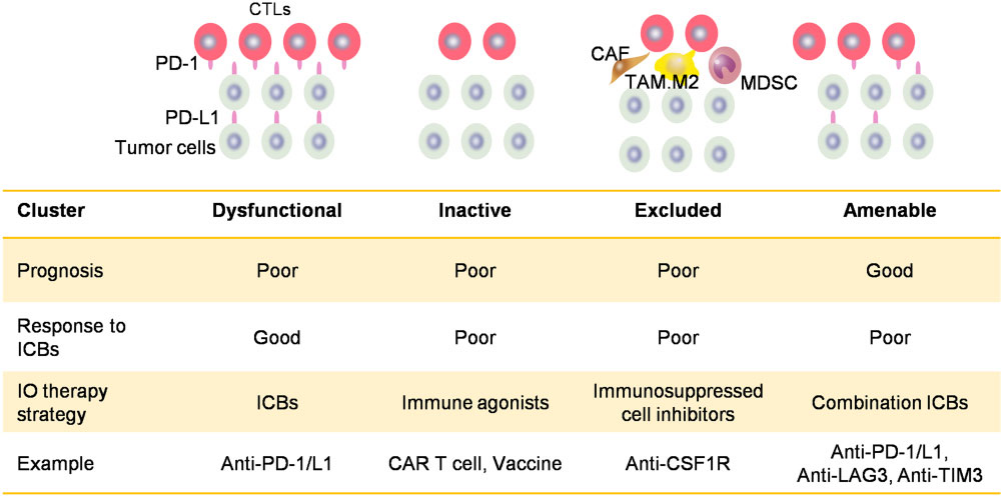
Proposed scheme for stratification of CRC patients for prognosis prediction and personalized immunotherapy.

Tumors were classified into four clusters based on the expression signatures of MDSC, CAF, TAM.M2, CTL, and PD-L1: immune-dysfunctional, immune-inactive, immune-excluded, and immune-amenable (Figure 6). The immune-dysfunctional cluster was characterized by both a high level of CTLs infiltration and PD-L1 expression, which lead to an impaired antitumor immune response with dysfunctional T-cells. The immune-inactive cluster had inadequate CTLs activation. The immune-excluded cluster was enriched by immune-suppressive cells that can hinder the CTLs from infiltrating into the tumor sites and promote tumor malignancy. The immune-amenable cluster showed a high level of immune activation and moderate dysfunction and exclusion. Patients in various clusters might respond to different treatments. The dysfunctional cluster has the potential to benefit from immune checkpoint blockades (ICBs); the immune-excluded cluster needs inhibitors to relieve T-cells from immunosuppressive cells; the immune-inactive cluster needs immune agonists or other methods to restore T-cell activation.

## Discussion

In this study, we integrated the signatures of CTL, MDSC, CAF, TAM.M2, and PD-L1 to obtain four clusters with distinct features. The immune-amenable cluster was predicted to have the best prognosis among all clusters and this was confirmed using survival data of both the TCGA and the GSE39582 cohorts. The immune features of the other three clusters also provided some clues as to how we could better manage different subtypes by tailoring treatment strategies for different tumor microenvironments. The immune-dysfunctional cluster had the potential to benefit from ICBs, whereas the immune-inactive cluster and immune-excluded cluster might need immune agonists and immune-exclusion inhibitors to boost immune response, respectively.

There are multiple steps involved in the generation of an immune response against tumor cells, including antigen presentation, dendritic cell maturation, T-cell activation and expansion, T-cell migration, and prolonged CTL activity [1]. Disruption of any single step may lead to escape from immune surveillance by tumor cells. Patients may be grouped into subtypes based on different TIMEs. In 2009, on the basis of the density of CD3^+^ and CD8^+^ T-cells at the tumor center (TC) and invasive margin (IM), four types of TIME models were first proposed [21]: cold tumors (low levels of CD3^+^/CD8^+^ at both TC and IM), altered excluded tumors (high levels of CD3^+^/CD8^+^ at IM but very little at TC), altered immunosuppressive tumors (low levels of CD3^+^/CD8^+^ at IM and TC), and hot tumors (high levels of CD3^+^/CD8^+^ at both TC and IM). Sanmamed *et al.* [22] proposed another popular preliminary TIME classification in 2018, in which tumors may be categorized based on their likelihood to respond to immunotherapy: type I (PD-L1-/TIL-, like cold tumors), type II (PD-L1^+^/TIL^+^, like hot tumors, anti-PD-1/L1 therapy), type III (PD-L1^−^/TIL^+^, like alter tumors), and type IV (PD-L1^+^/TIL^−^, like altered tumors). Our results supported the concept of patient classification based on TIME (Figure 6). As described above, the immune-inactive cluster was similar to cold tumors, with low levels of TIL signatures and PD-L1, and is therefore considered immune-ignorant because immune cells do not accumulate at the tumor site. The immune-excluded cluster was similar to altered excluded tumors, with low TIL but high T-cell-exclusion signatures. There are three major suppressive cells available to evaluate immune exclusion: MDSC, CAF, and TAM.M2. TAM.M2 activates the production of CAF and CAF in turn upregulates the expression of S100A8/9 in the myeloid cells that then differentiate into MDSC or TAM.M2 [23]. These immunosuppressive cells can hinder the CTL from infiltrating into the tumor sites and promote tumor malignancy [3]. The dysfunctional cluster had significantly higher CTL infiltration than the immune-amenable cluster, which is usually predictive of better immune responsiveness and thus better prognosis. However, the dysfunctional cluster also showed the highest PD-L1 expression, which can bind to PD-1 on the surface of T-cells and inhibit immune activity [24]. The mRNA of *PD-L1* is commonly found in various normal tissues [24] and the expression of PD-L1 on the cell surface can be induced by interferon gamma (IFNs) released by immune cells [25]. Therefore, the tumor-site-specific activation of the PD-1/PD-L1 pathway is determined by localized induction of PD-L1 by IFNG, which indicates that higher CTL releasing IFNG and higher expression of PD-L1 are signs of impaired antitumor immune response caused by dysfunctional T-cells [22]. In this manner, the dysfunctional cluster had weaker immune activity than the immune-amenable cluster. Taken together, compared with the other three clusters, the immune-amenable cluster had stronger antitumor immune activity and better OS.

We also compared the four clusters with the CMS reported by an international consortium [13, 17]. The immune-dysfunctional cluster had a high proportion of CMS1 (called MSI-like subtype) and CMS4 (called mesenchymal subtype). CMS1 was characterized by the highest expression of genes specific for cytotoxic lymphocytes and PD-L1/PD-L2 genes and CMS4 by massive tumor infiltration by both CD8^+^ T-cells and CAF [13], which are consist with the features of the immune-dysfunctional cluster with high T-cell infiltration, T-cell dysfunction, and CAF. CMS2 (called canonical subtype) and CMS3 (called metabolic subtype) are characterized by poor infiltration by immune cells [13], which are enriched for the immune-inactive cluster. The immune-excluded cluster is composed of 31.5% CMS2 and 42.1% CMS4, whose immune context integrated the low T-cell activation of CMS2 and the high CAF of CMS4 [13]. For the immune-amenable cluster, the ratios of the four CMSs were relatively even. Consistently with the CMS prognosis, the patients with cluster 1 tumor enriched with CMS1 and CMS4 had shorter OS than the patients with cluster 2–4 tumors. These results suggested that immune clustering based on the dynamics between immune activation and suppression may accurately predict prognosis.

Given the concept of ‘normalization cancer immunotherapy’ proposed by Dr Lieping Chen, in which the stress is placed on restoring antitumor immune competency rather than enhancing it according to specific TIME subtypes [22], our clustering strategy has the potential to guide personalized immunotherapy (Figure 6). The dysfunctional cluster, like hot tumors, has the potential to benefit from ICBs; the immune-excluded cluster, like the altered tumors, needs inhibitors such as anti-CSF1R to relieve T-cells from immunosuppressive cells [26]; the immune-inactive cluster, like the cold tumors, needs immune agonists or other methods (e.g. STING agonist, CAR-T-cell, and radiotherapy) to restore T-cell activation [26]. Our notion was recently supported by a phase 1b trial showing that the combination of regorafenib and nivolumab produced a 33% object response rate (ORR) in MSS CRC patients [27]. Regorafenib is known to decrease macrophage accumulation in murine models [28]. Furthermore, the prevalence of the immune-excluded cluster in MSS/MSI-L patients was 26%, which is close to the ORR in the above clinical trial [28].

In subgroup analyses, there was no significant difference in OS between patients with stage I and stage II CRC; however, immune clustering could identify the patients with poor prognosis in a stage I/II subset, suggesting that the immune clustering may serve as a complementary approach to pathologic staging for prognosis prediction. Besides, with the limited number of patients with a MSI-H phenotype or those having received adjuvant treatment, immune clustering was only found to be associated with OS within MSS/MSI-L patients or those not treated with adjuvant therapy. Taken together, for early-stage MSS patients without adjuvant treatment, our strategy of immune clustering may help to identify patients with poor prognosis who may benefit from adjuvant treatment after surgery.

Our study classified CRC patients based on the signatures of T-cell activation, dysfunction, and exclusion, which supports the notion of personalized immunotherapy according to specific TIMEs. There are several limitations of our study. First, the cluster analysis is qualitative, not quantitative. Second, although the TIDE algorithm makes it possible to assess signatures of TIME using mRNA expression, it is still necessary to validate the signatures with multiple immunohistochemical (mIHC) or immunofluorescence with regard to the density of various cell types in TC or IM. A validation study to explore the TIME using mIHC is currently under way. Third, detailed clinical information available in public databases was limited, especially for information on treatment, and this might have introduced bias to our analyses.

## Conclusion

In conclusion, four clusters with distinct immune contexts were identified among CRC patients: an immune-dysfunctional cluster, an immune-inactive cluster, an immune-excluded cluster, and an immune-amenable cluster. This subtyping strategy may serve as a prognosis predictor to accurately stratify patients with different outcomes. Its application in predicting immunotherapy efficacy also warrants further exploration in the future.

## Supplementary data

Supplementary data is available at *Gastroenterology Report* online.

## Authors’ contributions

Y.Q.T., J.Z., B.W., and J.H. designed this study. T.F.C., M.L.H., X.C.Z., J.Z., and Y.Z.Z. analysed the data. G.Q.W., Y.Z., and S.L.C. were major contributors in writing the manuscript. All the authors participated in the interpretation of data and in writing the manuscript. All authors read and approved the final manuscript.

## Funding

This work was supported by the National Natural Science Foundation of China [Grant No. 81972885] and Guangdong Basic and Applied Basic Research Foundation [Grant No.2018A0303130309].

## Supplementary Material

goaa045_supplementary_data
