# Supplementary figures and images for: The tumor immune microenvironment transcriptomic subtypes of colorectal cancer for prognosis and development of precise immunotherapy
Source: Gastroenterol Rep (Oxf). 2020 Sep 14;8(5):381–9. doi: 10.1093/gastro/goaa045 (PMC7603874; doi:10.1093/gastro/goaa045)

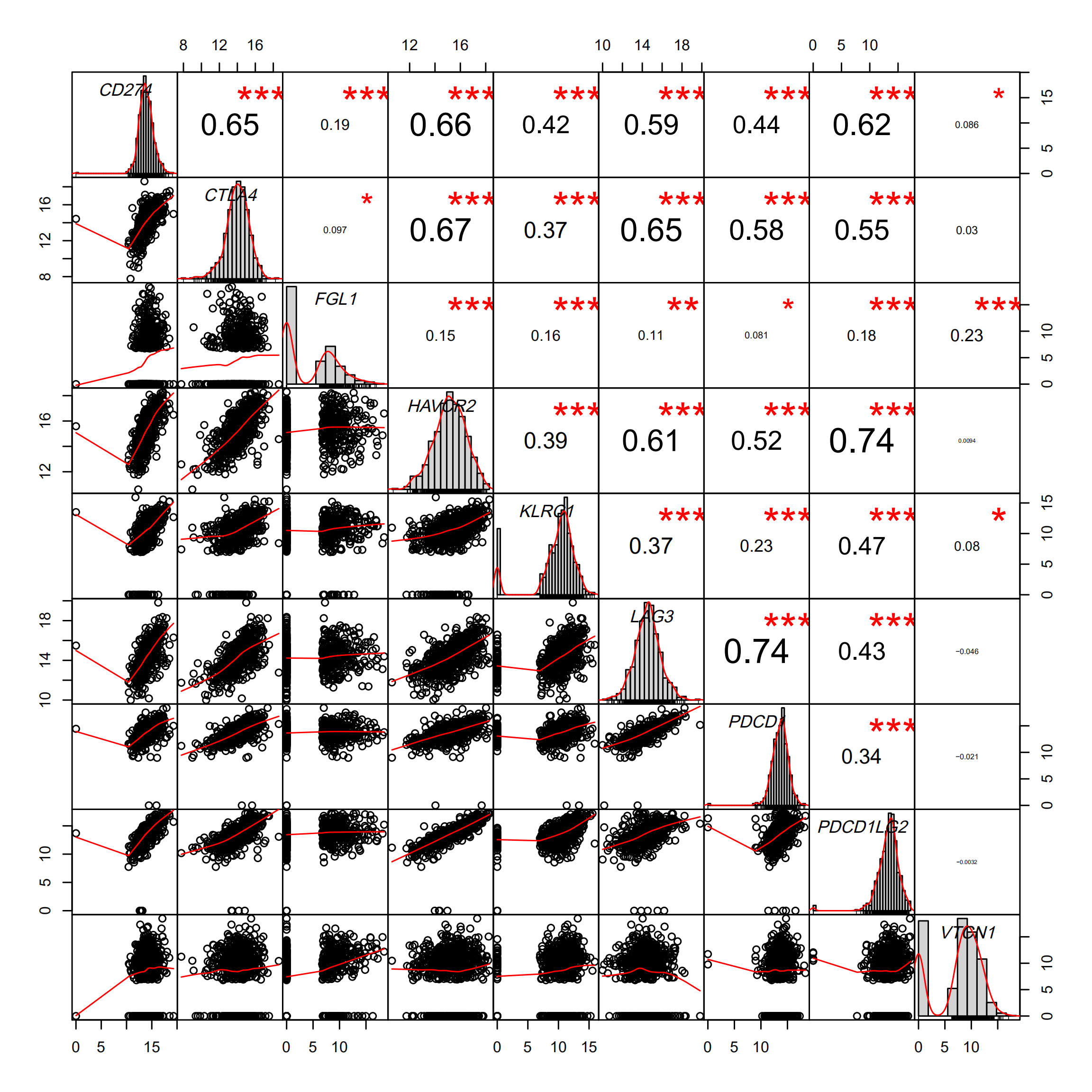

Supplement: goaa045_supplementary_data [file goaa045_supplementary_data.png]
